# Evidence for perceptual learning with repeated stimulation after partial and total cortical blindness

**DOI:** 10.2478/v10053-008-0099-8

**Published:** 2012-02-03

**Authors:** Ceri T. Trevethan, James Urquhart, Richard Ward, Douglas Gentleman, Arash Sahraie

**Affiliations:** 1 Vision and Attention Laboratories, School of Psychology, University of Aberdeen, UK; 2 Richard Ward Opticians, Odiham, Hampshire, UK; 3 Centre for Brain Injury Rehabilitation, Royal Victoria Hospital, Dundee, UK

**Keywords:** blindsight, visual field training, Neuro-Eye Therapy, spatial frequency, feedback, perceptual learning

## Abstract

Lesions of occipital cortex result in loss of sight in the corresponding regions of visual fields. The traditional view that, apart from some spontaneous recovery in the acute phase, field defects remain permanently and irreversibly blind, has been challenged. In patients with partial field loss, a range of residual visual abilities in the absence of conscious perception (*blindsight*) has been demonstrated (Weiskrantz, 1986). Recent findings (Sahraie et al., 2006, 2010) have also demonstrated increased visual sensitivity in the field defect following repeated stimulation. We aimed to extend these findings by systematically exploring whether repeated stimulation can also lead to increased visual sensitivity in two cases with total (bilateral) cortical blindness. In addition, for a case of partial blindness, we examined the extent of the recovery as a function of stimulated region of the visual field, over extended periods of visual training. Positive auditory feedback was provided during the training task for correct detection of a spatial grating pattern presented at specific retinotopic locations using a temporal two alternative forced-choice paradigm (Neuro-Eye Therapy). All three cases showed improved visual sensitivity with repeated stimulation. The findings indicate that perceptual learning can occur through systematic visual field stimulation even in cases of bilateral cortical blindness.

## Introduction

Visual field loss following brain injury is common, affecting approximately 30% of stroke cases (Pambakian, Mannan, Hodgson, & Kennard, 2004). Previously it was assumed that a unilateral post-geniculate lesion resulted in an area of total and permanent blindness in the corresponding region of visual field (Holmes, 1918). Similarly, a bilateral post-geniculate lesion would result in complete and permanent blindness. Since the 1970s, a substantial body of evidence for the ability to detect, localise, and discriminate a range of visual stimuli within blind areas of visual field in human subjects has emerged. Much of the evidence for residual visual abilities comes from psychophysical testing aimed at bridging the gap between human and other animal studies, by avoiding verbal commentaries and instead, asking the subject to choose, if necessary by guessing, amongst alternatives. For example, the participant may be asked to choose in which of two time intervals (signalled by audio markers) a visual stimulus was presented within the blind area of visual field. Such abilities may occur without any or in some instances with only a limited conscious awareness of the event whilst patient’s denying the experience of seeing the visual targets.

*Blindsight* is traditionally defined as visual capacity in the field defect in the absence of acknowledged awareness. There are occasions, however, when a patient reports some awareness of visual events, but yet denies any experience of seeing. It is important to note that those with partial vision loss retain normal visual capacities within their sighted field, which they can use to compare with experiences within their blind field. Visual capacities within the field defect with some awareness, but in the absence of acknowledged seeing is referred to as *blindsight type II* (Weiskrantz, 1997). The incidence of blindsight amongst hemianopic samples tested varies substantially (5% in Marzi, Tassinari, Agliotti, & Lutzemberger, 1986; 0-60% in Stoerig, 1987; 20% in Blythe, Kennard, & Ruddock, 1987; and 80% in Sahraie et al., 2003), which is not surprising given the range of techniques and stimulus parameters used.

The spatial properties of channels mediating blindsight were first examined in detail in a well documented case (G.Y.; see Barbur, Harlow, & Weiskrantz, 1994). The findings showed that in addition to a luminance-flux channel responsible for detection of brightness, the spatial mechanisms leading to blindsight had specific spatial and temporal tuning properties. The spatial channel appeared to have band-pass characteristics with peak sensitivity at approximately 1 cycle/°.The temporal sensitivity was also characterized by a band-pass, being preferentially sensitive to temporal variations between 5-20 Hz. These findings were confirmed in a larger cohort study for spatial characteristics (Sahraie et al., 2003), although in some patients the spatial channel was low-pass with peak sensitivity again at or below 1 cycle/°. The temporal characteristics of the spatial channel were also extended to a larger cohort (Sahraie, Trevethan, & Macleod, 2008).

### Visual rehabilitation of partial visual field defects

During the last three decades, some issues relating to intervention-induced change in visual sensitivity have been controversial (for reviews, see Huxlin, 2008; Sahraie, 2007), nevertheless there is increasing evidence for intervention-induced sensitivity change in patients with partial visual field defects.

The *compensatory strategies* refer to those where patients are trained to modify their eye-movements to explore their environment. In an animal model, Mohler and Wurtz (1977) demonstrated changes in saccadic localisation ability following systematic training in two monkeys. Using a similar saccadic localisation task with hemianopic patients, Zihl and von Cramon (1985) reported a similar pattern of results. Different groups have since extended these findings to other hemianopic patients using similar visual search tasks (Kerkhoff, Munßinger, & Meier,1994; Lane, Smith, Ellison, & Schenk, 2010; Mannan, Pambakian, & Kennard, 2010; Pambakian et al., 2004).

*Restitution techniques* refer to those where the experimenters have aimed to increase the visual sensitivity within the field defect using repeated stimulation paradigms. Using small dot stimuli in a detection task, Kasten, Wust, Behrens-Baumann, and Sabel (1998) demonstrated that repeated stimulation could result in improved detection of targets within the sighted/blind boundaries. The technique has been effective in showing improvements in large groups of patients (*N* = 307 in Mueller, Mast, & Sabel, 2007). A common factor across all of these approaches was the presentation of visual stimuli at blind/sighted border, where visual sensitivity was reduced, rather than stimulation deep within the visual field defect. Although questions regarding possible eye movement inaccuracies in relation to assessment of the efficacy of both compensatory and restorative rehabilitation interventions have been raised (Balliet, Blood, & Bach-y-Rita, 1985; Reinhard et al., 2005), there is further evidence that recovery is independent of eye movement strategies (Kasten, Bunzenthal, & Sabel, 2006). There is also a range of converging evidence from a number of different laboratories for recovery of functions using flickering targets (Henriksson, Raninen, Nasanen, Hyvarinen, & Vanniet, 2007; Julkenen, Tenovue, Jaaskelainen, & Hamalainen, 2003), flashing grating patterns (Sahraie et al., 2006, 2010), and moving random-dots (Huxlin et al., 2009). The findings are often attributed to visual plasticity in the injured brain (Huxlin, 2008; Sabel, 2008). Importantly, these findings include evidence for changes in sensitivity in areas deep within unilateral visual field defects, with some evidence that provision of positive feedback can accelerate recovery (Sahraie et al., 2010). In all three cases showing recovery with training, the rate of recovery was higher for stimuli presented within the borderline/transition zone compared to areas deeper within the field defect. This finding raises the question of whether any changes in visual sensitivity at locations deep within the visual field are contingent upon a change in neighbouring areas within the sighted field (Sahraie et al., 2010).

It is perhaps not surprising that the published research into intervention-induced changes in visual sensitivity has almost exclusively focused on cases with unilateral field loss, particularly as this is the most common type of field defect (Zhang, Kedar, Lynn, Newman, & Biousse, 2006). Cases with this type of field loss are also more likely to have preserved physical and cognitive abilities required to complete training tasks, reflecting the usually less severe brain injury (Zhang et al., 2006). Focusing on these types of cases also provides the advantages of greater opportunity for systematic study due to better attention span or concentration as well as possibility of obtaining detailed perimetric visual fields to track changes in sensitivity.

### The possible contributions of studying cases with total cortical blindness

Previous evidence for residual visual abilities in cases of total cortical blindness has been restricted to luminance-based stimuli (e.g., Brindley, Gautier-Smith, & Lewin, 1969). It is of particular interest to explore whether successful discrimination of stimuli with the spatial and temporal characteristics which have elicited residual vision and increased sensitivity with training in cases with partial blindness, can also be effective in those with total cortical blindness. Exploring whether increased sensitivity occurs with training in bilateral cases is also important as bilateral cortical blindness rules out the possible role of artefacts such as eye movement strategies, stray light, and the possible use of the sighted field (Campion, Latto, & Smith, 1983; Cowey, 2004). Investigating the effects of repeated stimulation on cases with bilateral cortical blindness is also important in relation to the mechanisms for recovery as the sighted/blind border interactions are absent in total cortical blindness. Nevertheless, investigation of such bilateral cases is necessarily complex as a result of the greater extent of brain injury, reliance on less rigorous clinical methods of visual assessment in the absence of perimetric data, and other related factors such as fatigue and shorter concentration span (Gaber, 2010).

Here we report two sets of investigations. Firstly, we have examined whether repeated stimulation can increase sensitivity in two cases with total cortical blindness. Secondly, we provide preliminary evidence that the recovery of function is restricted to the stimulated location in a patient with partial blindness.

## Materials and Methods

### Bilateral case B1

This 31-year old male sustained a high cervical cord injury (C2 level) as a result of an accident in 2004. His injuries caused immediate (and persisting) tetraplegia, as well as cardiorespiratory arrest with cerebral hypoperfusion and anoxic-ischaemic brain injury, resulting in bilateral occipital infarcts.

His initial cognitive function was severely affected but improved over the first few months. He is able to interact with others on a day-to-day basis, to make choices, and to communicate his wishes clearly. Formal neuropsychological assessment on two occasions showed some improvements in information processing speed and attention over the first 2 years since his injury. He now ventilates spontaneously for up to 18 hr a day, but still requires overnight ventilation.

He is functionally completely cortically blind and both initial and subsequent bedside clinical assessments demonstrated no light perception. Because of his injuries it was not possible to complete formal perimetric testing and MRI (magnetic resonance imaging) is also contraindicated.

### Bilateral case B2

This 23-year old female university graduate was involved in a bicycle versus truck collision. A tear in the right atrium caused a cardiac tamponade which required surgical intervention. She also had a liver laceration, extensive fractures of the ribs and upper and lower limbs, a punctured lung, and a severe traumatic brain injury with cerebral and cerebellar contusions and a skull base fracture. As a result of the cardiac arrest and blood loss she had hypoxic-ischaemic brain damage that led to bilateral occipital infarction.

B2 underwent an extensive period of inpatient rehabilitation in a specialist centre and then community-based rehabilitation that focused on improving her mobility, functional independence in the home, safety, and cognition. She made progress but still has major cognitive, language, and physical impairments. However, she is able to make and communicate choices. Clinical assessment and subjective reports did not reveal any intact areas of visual field.

Because of the nature of their injuries it was not possible for B1 and B2 to obtain MRI scans.

### Unilateral case U1

U1 is a 67-year old male who suffered anoxia following heart valve failure in September 2005. As a result, he had a left hemianopia which persisted 6 months later when he started the visual training. CT (computed tomography) scans taken shortly after the anoxic episode showed no signs of brain abnormality. There were no other perceptible sensory deficits or motor impairments. Prior to the start of the visual training task and during the training, U1 underwent multiple sessions of hyperbaric exposure.

### Visual training program

The principles behind the Neuro-Eye Therapy have been previously detailed elsewhere (Sahraie et al., 2006, 2010). In brief, it relies on repeated stimulation at specific retinal locations, using grating patterns presented on a home computer. The sinewave grating patterns have the same space-averaged luminance as that of the background (37 cd/m^2^), with spatial and temporal frequencies (1 cycle/°, 10 Hz) matching the optimum characteristics for the residual visual processing. Instead of passive viewing, the patients are asked to pay attention to their blindfield while fixating on a specific point on the screen. Two consecutive time intervals are denoted by auditory signals and during one of the intervals, a grating pattern is presented. The remaining interval does not include a visual target and only the background is present. A final auditory signal indicates the end of the second time interval, following which the patient is asked to respond using a button press. Patients are asked to choose, if necessary by guessing, the time interval (first or second) where a visual target was presented and to indicate their choice by pressing one of the two response buttons. B1 verbally reported the target interval and a carer pressed the buttons, B2 used manual key presses. The chance performance for a two alternative forced-choice task is 50%, and the initial target contrast is set at 90% (maximum contrast). For patients with good concentration span, there are 50 pre-sentations at each of three stimulus locations. When patients perform significantly above chance ( 86% correct) at three consecutive training sessions, the target contrast is reduced by 10%. This is to ensure that the task difficulty remains high throughout the training. If the perfor-mance falls substantially ( 64%) the target contrast is increased by 5%. The process is repeated until either the performance increases or the target contrast reaches 90%. The upper limit was adjusted if less than 50 presentations are shown at each location (see below) and lower limit removed. Grating patches were presented at three predefined locations within the field defect. For those with partial sight loss, the circular patches are 6° in diameter in order to allow for localised stimulation within the field defect. For cases of total cortical blindness, a number of issues are worth emphasizing. Although patients could be instructed to maintain their gaze direction at the centre of the display screen, there are no reliable techniques to ensure steady fixation other than repeated instruction for holding steady gaze. In addition, large target size and short viewing distance can ensure maximum exposure.

**Figure 1.**
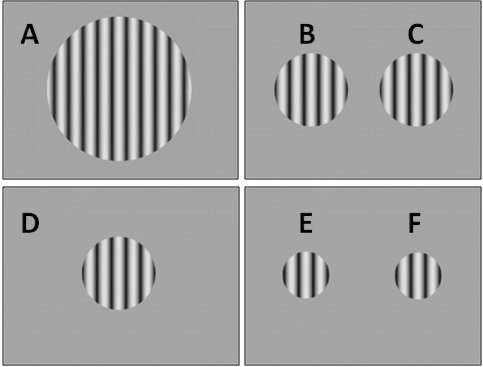
Schematic representation of targets shown on the display screen to the bilateral cases. Letters A-F on this figure refer to the corresponding letters on the data plots in Figures 2 and 3.

For B1 and B2, three different gratings were presented during the Block 1 trials, one large (50° diameter), centrally presented grating, one smaller (25° diameter) grating presented in the centre of the right visual field and one (25° diameter) presented in the left visual field (see Figure 1). During Block 2 (B1 only), the diameter of the large grating was 25° and the smaller gratings were 11.5°. The duration of each training session had to be kept short due to fatigue and therefore, for B1 there were 20 presentations of each stimulus during a training session (a total of 60 presentations), whereas B2 was shown 30 presentations of each stimulus during a training session (a total of 90 presentations).

After each trial, B1 responded verbally to indicate in which one of the two intervals he detected the grating (“first” or “second”), and a carer operated the response buttons on his behalf due to his tetraplegia. B2 reported the interval containing the grating by pressing one of two response buttons (designated as “Interval 1” and “Interval 2”). The stimulus presentations were self-paced and the program paused if there were no responses. The program also enforced a 5-min break at the mid-point of each session. Auditory feedback was provided in the form of a high tone for a correct response. The stimulus contrasts were kept constant during a training session but were modified in subsequent sessions depending on the performance as follows. Initial target contrast was 90%. For B1 (block one), if performance was equal or better than 17 out of 20 (85% correct) in three consecutive sessions then the contrast for that stimuli was reduced by 10% for the next training session. The first block of testing took 12 months for B1 and 2.5 months for B2. After an 18 month gap, a second block of training was conducted. The second block of testing was conducted over a 6 months period (B1 only) where the upper threshold was set to 16 out of 20 (80% correct). Therefore, good performance led to reduction in contrast, and the initial target contrast was set at 60%. For B2, the upper threshold for contrast reduction was set to 25 out of 30 (83% correct).

**Figure 4.**
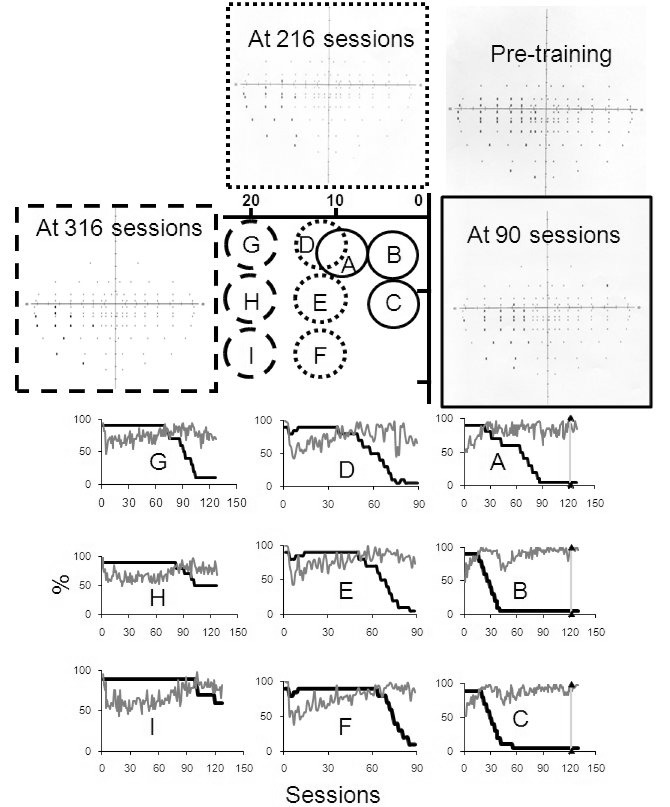
At the centre of the figure a schematic representation of stimulus locations used during training is depicted. Initially the patterns were shown at locations A-C. The detection performance (grey lines) and target contrasts (dark lines) for each training session are also plotted. Detection performance improves with time and remains high even when the stimulus contrast is reduced. When all pattern contrasts reached 5% the block of testing terminated and the suprathreshold visual fields were obtained. The patterns were then moved leftward during subsequent blocks of training and the pattern contrasts were reset to 90%. Plots D-F and G-H represent the detection task performance in the second and third blocks of training.

For U1, there were three blocks of training and the positions of the targets are shown at the centre of Figure 4. As the performance improved, the three target stimuli were shifted deeper within the field defect as indicated. The upper and lower limits for contrast manipulations were set as per the standard Neuro-Eye therapy (Sahraie et al., 2006, 2010). That is, a reduction in contrast of 10% after 43 out of 50 (86%) correct and an increase of 5% contrast if the performance fell to or below 32 out of 50 (64%) correct. The three blocks of training included 129, 80, and 127 sessions, respectively. Binocular (Esterman) visual fields were obtained using a Humphrey’s visual field analyser, before the start of the training and after U1 had completed 90, 216, and 316 training sessions in total.

## Results

**Figure 2.**
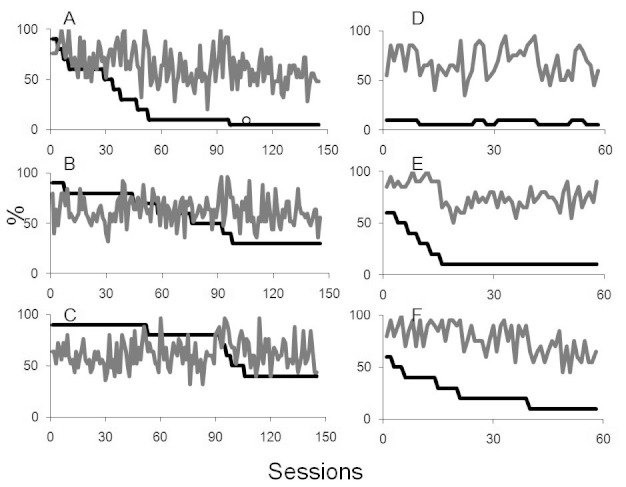
The left hand panels relate to first block of training in bilateral case B1, with plots A, B, and C representing contrast levels and detection scores for centrally presented targets, and targets presented to the right and left respectively. D, E, and F plots the corresponding data for the second block of training. In all plots, target contrasts are shown in dark lines and the grey lines plot the detection score.

Figure 2 (Panels A, B, & C) shows the results for B1 when a large central and two smaller targets were presented on the right and the left, respectively. B1 carried out a total of 145 sessions of training during block one (a total of 8,700 individual trials). As shown in Panels A, B, and C, B1’s performance was variable. There were days that he performed very well and others when he performed at chance level. It is important to note that there were short periods in which he was unwell and was unable to participate in the training program. Overall, in response to the 50° diameter, centrally presented target, B1’s peak detection on occasions was perfect, but overall he averaged 55% for a 5% contrast target (Figure 2, Panel A). The mean detection for the target presented in his right visual field, peaked at 92% correct detection, but averaged at 58% for a 30% contrast target (Figure 2, Panel B). B1’s peak performance for the target presented in his left visual field was 96% detection of a 40% contrast target, but averaged 60% overall (Figure 2, Panel C).

We also attempted to obtain measures of detection performance before and after Block 1 of training in B1 using a temporal two-alternative forced-choice (2-AFC) detection technique. When B1 was tested initially with 60 trials of the 2-AFC task, using a 40% contrast grating, he performed at chance (48% correct) at detecting the presence of the grating and was aware of 8% of target presentations but none of the targets he reported awareness of were actually correctly identified (0% correct and aware discrimination). When B1 was re-tested after completion of the first training block with the same stimuli, he was 78% correct (*p* < .05 binomial test) at detecting the presence of the target with 73% reported awareness. Crucially, he was 81% correct at detecting the presence of the grating when he reported experiencing awareness (36 out of 44 trials, *p* < .05 binomial test).

We repeated the measurements, 14 months after the end of the first block of training. For a 40% contrast stimulus, he was at chance (60% correct) at detecting the stimulus. He reported awareness of 30% of presentations but was correct and aware of only 17% of presentations. However, when he was tested with a 90% contrast stimulus he was 95% correct at detecting the presence of the target and 75% correct and aware, indicating that although detection was possible, there was a reduction in sensitivity on the day the test was conducted.

B1 completed a second block of training. This block comprised 58 sessions with a total of 3,480 individual trials, the results of which are shown in Figure 2 (Panels D, E, & F). For the large, centrally presented target, B1’s mean detection performance for the 5% contrast central stimulus was 60% correct (Figure 2, Panel D), and for 10% contrast right target 71.6% correct (Figure 2, Panel E). The mean detection for 10% contrast target presented on the left was 63.4%.

**Figure 3.**
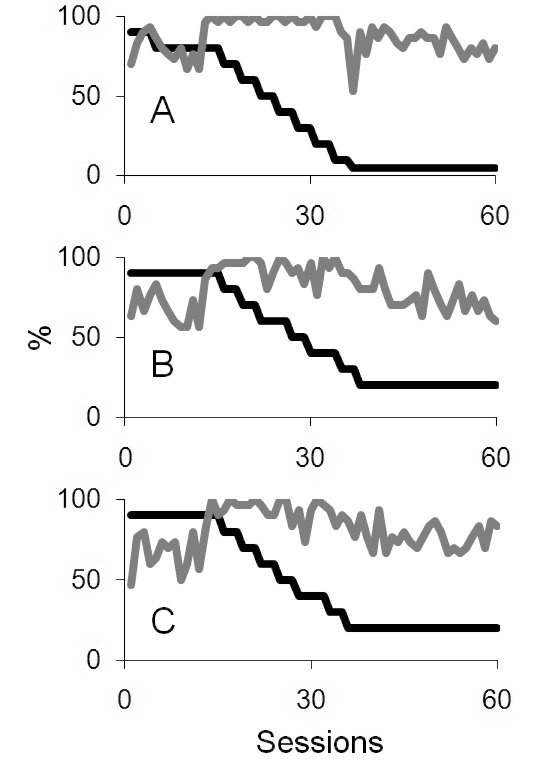
A, B, and C represent the target contrasts and detection scores for targets presented at the centre, left, and right of the display for the bilateral case B2. In all plots, target contrasts are shown in dark lines and the grey lines plot the detection score.

Figure 3 shows the results of the training program for B2. Results for the large, centrally presented stimulus are plotted in Figure 3 (Panel A). Initially, B2 was 70% correct at discriminating the high (90%) contrast target. Following 60 training sessions (a total of 1,800 individual trials at that location), for a 5% contrast stimulus, B2 was 80% correct at detecting the presence of the stimulus. Figure 3 (Panel B) shows her detection of the smaller target (25° diameter) presented in the right of her visual field, revealing a shift from initial detection of a 90% contrast target of 63% correct to being able to detect a 20% contrast target 60% correctly. Panel C of Figure 3 shows detection of the 25° diameter target presented in the left visual field. She was initially 47% correct of the 90% contrast target and following 60 training sessions, she was 83% correct of a 20% contrast target.

Unfortunately, due to unforeseen circumstances, psychophysical data could not be collected for B2 at the end of the block, but overall, data plotted in Figure 3 scored consistent with high detection, with decreasing target contrasts, indicating increased visual sensitivity.

Figure 4 summarises the findings for U1. The fixation is indicated by coordinate 0 at the centre of the figure and 10 and 20° eccentricities in the left hemifields are also denoted. Three grating patches were presented near the fixation and they were subsequently moved deeper within the field defect. The detection scores (grey lines) in a temporal 2-AFC paradigm for presentation at each eccentric location are also plotted, together with the stimulus contrast at each training session (dark lines). Overall, the detection scores improved after a few sessions and if they were consistently above the upper limit (86% correct), the contrast was lowered in steps of 10%. Binocular Esterman visual fields were also plotted before the training and on three other occasions after 90, 216, and 316 sessions. The fields showed gradual improvements and a reduction in the extent of the field defect. Importantly, the improvements were linked to the stimulated area, indicating that the recovery of sensitivity is location specific. U1 took a one month break from training after 112th session, during the first block of testing, indicated by a vertical line in the data panels. As depicted in the data plots, this break did not affect performance in subsequent sessions.

## Discussion

Data for two cases with cortical blindness following bilateral occipital lesions have been presented. Patients underwent repeated stimulation of vision using large patches of spatially and temporally modulated stimuli, previously shown to increase visual sensitivity in those with partial sight loss after brain injury. Data for both cases indicate that visual sensitivity, as measured using a forced-choice detection task, can increase following repeated exposure. In addition, we have systematically stimulated the visual field of a patient with unilateral visual loss, using a similar technique. The findings show that the increased sensitivity is specific to the stimulated region of the visual field and does not lead to better performance in a non-stimulated area.

Evidence for the existence of residual visual processing abilities in humans following brain injury affecting striate cortex has recently increased and grown to include converging evidence from a variety of methodologies. The findings have been accompanied by a growing interest in the possibilities for training aimed at recovery of function. There is considerable evidence from animal studies that changes in visual sensitivity can occur with training following ablation of striate cortex (Cowey & Weiskrantz, 1963; Mohler & Wurtz, 1977). Studies investigating rehabilitation of visual field defects in humans have focused on unilateral visual field defects and have usually employed a strategy of presenting visual stimuli in the boundary between a blind area of visual field and an area with amblyopic or normal vision (e.g., Kasten et al., 1998; Zihl & von Cramon, 1985). However, recent evidence has demonstrated the existence of a narrowly tuned channel of processing within the field defect with specific spatial and temporal tuning properties (Barbur et al., 1994; Sahraie et al., 2003, 2008). Stimulation of the blind field using such specific visual targets matching the channel properties have lead to increased detection ability within the field defect (Sahraie et al., 2006, 2010). The results from two cases with bila-teral cortical blindness reported here extend these findings.

There are a number of points that need to be emphasised in relation to both cases. Firstly, the learning to detect targets appears to be a slow process. B1 undertook 12,180 trials (8,700 and 3,480 in Blocks 1 and 2, respectively) and showed substantial improvement in detection ability. B2 was only able to complete 5,400 trials, but nevertheless, still showed improvements in sensitivity. Prolonged and extended testing sessions are a challenge for cases where there is extensive brain injury. The extent of any sensitivity change that would be expected is also limited and may be confined to detection of some visual transients. For unilateral vision loss, previous work suggests that a more positive prognosis may be expected (Huxlin, 2008; Huxlin et al., 2009; Stoerig, 2008). U1 was able to complete 50,400 trials. This prolonged and systematic exposure led to both higher detection ability, and improved binocular visual fields.

The mechanism for recovery is not clear. In the presence of extensive brain injury, the recovery can be as a result of visual information being processed via alternative pathways, or the repeated exposure can lead to formation of new pathways. The former is a strong possibility as in addition to geniculo-striate projection there are nine other candidate routes where the visual information by-passes the striate cortex (Weiskrantz, 1986). Contra-lesioned hemisphere may also receive information from the field defect via sub-cortical routes, mainly the Collicular callosal connections. Leh, Mullen, and Ptito (2006) demonstrated correlation between the behavioural findings of sighted/blind field interactions and the existence of strong inter-hemispheric subcortical connections in hemispherectomized patients. The latter mechanism has been recently demonstrated in a hemianopic patient with early brain damage (Bridge, Thomas, Jbabdi, & Cowey, 2008). Using imaging techniques, Bridge et al. have shown the existence of stronger inter-hemispheric connections between extrastriate areas in one hemianopic patients compared to other healthy control cases.

Evidence for neuronal plasticity in normal observers comes from the findings that repeated stimulation/exposure can improve perfor-mance on a range of discrimination tasks, a phenomenon often referred to as *perceptual learning* (Fahle, Edelman, & Poggio, 1995; McKee & Westheimer, 1978). In particular, provision of feedback can accelerate learning (Herzog & Fahle, 1997). The body of evidence from previous reports in hemianopic patients and those reported here support the notion of visual plasticity and perceptual learning within the injured brain. Although the exact mechanisms underpinning the recovery of visual sensitivity in cases of total cortical blindness and those of unilateral field loss are not known, both above routes remain possible candidates. The improvements in detection performance appear to remain over relatively short time intervals. It would be of great interest to conduct a systematic study to exa-mine the long term changes in visual sensitivity, in the absence of any further training.
